# Evaluating human-centred design for public health: a case study on developing a healthcare app with refugee communities

**DOI:** 10.1186/s40900-021-00273-2

**Published:** 2021-05-30

**Authors:** Rebeccah Bartlett, Jacqueline A. Boyle, Jessica Simons Smith, Nadia Khan, Tracy Robinson, Rohit Ramaswamy

**Affiliations:** 1 Monash Centre for Health Research and Implementation – MCHRI, School of Public Health and Preventive Medicine, Monash University, Melbourne, Australia; 2 Public Health Leadership Program, Gillings School of Global Public Health, University of North Carolina at Chapel Hill, Chapel Hill, USA; 3 School of Nursing, Midwifery & Indigenous Health, Faculty of Science, Charles Sturt University, Bathurst, Australia

**Keywords:** Human-centred design, Design thinking, Refugee health, Evaluation

## Abstract

**Background:**

Australian women from migrant and refugee communities experience reduced access to sexual and reproductive healthcare. Human-centred design can be a more ethical and effective approach to developing health solutions with underserved populations that are more likely to experience significant disadvantage or social marginalisation. This study aimed to evaluate how well Shifra, a small Australian-based not-for-profit, applied human-centred design when developing a web-based application that delivers local, evidence-based and culturally relevant health information to its non-English speaking users.

**Methods:**

This study undertook a document review, survey, and semi-structured interviews to evaluate how well Shifra was able to achieve its objectives using a human-centred design approach.

**Results:**

A co-design process successfully led to the development of a web-based health app for refugee and migrant women. This evaluation also yielded several important recommendations for improving Shifra’s human-centred design approach moving forward.

**Conclusions:**

Improving refugees’ access to sexual and reproductive health is complex and requires innovative and thoughtful problem solving. This evaluation of Shifra’s human-centred design approach provides a helpful and rigorous guide in reporting that may encourage other organisations undertaking human-centred design work to evaluate their own implementation.

## Plain English summary

Australian women from non-English speaking migrant and refugee communities face reduced access to sexual and reproductive healthcare and many then go on to experience poor health outcomes as a result. There is an urgent need for new approach to improve access to healthcare for underserved communities, one that centres these women in the process of finding, developing and disseminating the solutions themselves. Human-centred design can be a more ethical and effective methodology in working with communities to develop these health solutions.

This study aimed to evaluate how well Shifra, a small Australian-based not-for-profit focused on improving access to healthcare for refugees and new migrants, undertook human-centred design approach when developing a Smartphone app that delivers local, safe and culturally relevant health information to non-English speaking Australians. The authors interviewed refugees, health and social sector experts and computer programmers involved in creating Shifra to evaluate how well they used human-centred design to achieve its goals. This evaluation found that Shifra’s approach was successful whilst also highlighting several important recommendations for improving collaborative efforts with refugee communities. These findings could help other projects also seeking to undertake an authentic community co-design process.

## Introduction

During 2017, Australia became home to 16,757 refugees [1]. Women and children, who comprised 87% [1, 2] of these new arrivals, face significant health challenges, including limited access to quality sexual and reproductive health (SRH) services [3, 4]. Poor SRH care has intergenerational consequences, affecting health and psychosocial outcomes for both mothers and their children [5]. Conversely, access to quality SRH services improves a number of health outcomes in women including prevention and management of high-risk pregnancies, reduction in unplanned pregnancies and abortions, reduction in obstetric complications, decreased anaemia and improved nutrition for both mother and baby [6, 7]. Despite these benefits, within Australia, women from migrant and refugee communities report less SRH awareness and experience reduced access to SRH-specific care as well as culturally-relevant support that could assist them to make evidence-based decisions about their own health and service utilisation [2]. A new approach to improve access to healthcare for underserved communities, particularly for women from refugee and migrant backgrounds, is needed, one that centres these women in the process of finding, developing and disseminating the solutions themselves.

Addressing public health problems through human-centred design (HCD) can be a more ethical and effective approach to developing solutions with underserved populations that are more likely to experience significant disadvantage or social marginalisation [8, 9]. HCD utilises multidisciplinary teams to approach the problem-solving process through three distinct phases: Inspiration, Ideation and Implementation [10]. HCD utilises “techniques which communicate, interact, empathise, and stimulate the people involved, obtaining an understanding of their needs, desires, and experiences, which often transcends that which the people themselves actually realised” [11]. Human-centred design, design thinking, co-design, co-production and co-creation are all terms that are often used interchangeably despite having nuanced differences in application and outcome. Each of these approaches focus on addressing complex problems and designing solutions with the end user communities (i.e. beneficiaries) [12, 13]. Design thinking is a specific set of stages within the HCD approach which help to guide problem solving teams through the whole experience as it diverges and converges the Inspiration, Ideation and Implementation phases in an iterative manner. Co-design, short for collaborative design is the process of design thinking steps that includes generative research (i.e. learning from end users) and development design (i.e. creating solutions with end users) [14]. This second, development design stage, is often termed co-production and together with co-design, these two stages form co-creation.

Studies have demonstrated promise that using HCD when developing health interventions can improve health outcomes for diverse populations [15–17] and that solutions developed using this approach result in increased uptake of services [18]; produce higher quality products and interventions; and that these products and interventions increased beneficiary satisfaction [19]. Importantly, this approach allows for the development of locally-driven, contextually-appropriate information that is crucial for meeting the health literacy needs of this population. However, a scoping review analysing 21 different studies for use of HCD in global health across various geographies and populations was unable to draw definitive conclusions about the effectiveness, because of the heterogeneity of implementation, application areas and contexts [20]. There remains a lack of understanding regarding how best to achieve and evaluate a successful HCD-driven solution [20] and how to destabilise power structures inherent to the HCD process itself. Maya Goodwill (2020) argues five different yet interrelated forms of power exist within the design process. These include privilege, access power, goal power, role power and rule power” [21]. These power differentials are present no matter how well-intentioned the design process is and there is a growing need to evaluate the implementation of programs that apply HCD principles, particularly when working with communities affected by the legacy of colonisation and systemic bias. Implementation evaluations not only assess a program’s deliverables against intended goals but also identify the strengths and weaknesses of a the implementation process, informing replication and efforts to scale [22]. To date, there are limited studies or reports detailing an implementation evaluation of an entire HCD project. Most studies address only one aspect of the design process such as planning [18, 23], prototyping [24] or assessing stakeholder engagement [25] but do not evaluate the entire approach. This study aimed to evaluate the HCD approach that Shifra, a small Melbourne-based not-for-profit focused on improving access to healthcare for refugees and new migrants, undertook in developing a web-based application to deliver local, evidence-based and culturally relevant SRH information to its users. Future papers will assess the relative success of the Shifra app in achieving its intended outcomes related to improving SRH literacy within non-English speaking refugee communities.

### Context

The Shifra web-app (herein simply referred to as an “app”), provides high quality, rights-based information on family planning, pregnancy and newborn health, sexuality and sexual health, as well as mental health, family violence and adolescent health. Written and video resources provide information on accessing health services in Australia and cover topics such as healthcare rights and responsibilities, accessing translating and interpreting services, public and private insurance, as well as clinic locations [26]. The app was originally designed for English and Arabic-speaking communities living in Melbourne and is in the process of being translated into other languages. As Shifra is committed to working in partnership with refugee and migrant communities to create products that are both ethical and sustainable [26], the team chose to apply a HCD approach, using design thinking methods to prototype and eventually develop its digital health intervention. Co-designers for the app included refugee end users, subject matter experts (SMEs) from different, partner organisations that focus on health for multicultural communities, user experience (UX) students, and computer programmers. Given the sensitive nature of the content for this app, SMEs were used to support and reinforce the refugees’ opinions when designs may be seen by some in the community as confronting. This happened in one instance, where a refugee end user noted that icons used to reference herpes were inappropriate. The designers questioned this, an SME was independently consulted in the same session and supported the initial opinion that the image was inappropriate and should not be used. Following this session, the designer’s supervisor was notified of the issue and the refugee who made the initial observation was debriefed and reassured that her opinion was valid and paramount to the development of a sensitive and quality app.

Local partnerships were integral to completing many of the design steps. In 2017, Shifra collaborated with an undergraduate UX class from Monash University’s School of Art Design and Architecture. A semester-long process to design a digital health solution to bridge the gap in refugees’ access to SRH services resulted in five prototypes. Shifra’s founder (RB) then selected two designs to combine and develop further in conjunction with the co-designers to incorporate end users’ values and cultural beliefs [27]. The Shifra team used a combination of design thinking approaches developed by IDEO, Stanford’s d-School and Mummah et al. (2016) [10, 24].

**Fig. 1:**
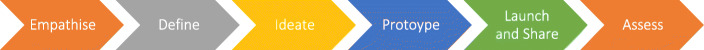
Design Thinking Process Adaptation for Shifra

IDEO’s Field Guide to Human Centred Design [10] and the Stanford d-School’s Process Guide influenced the development of the Empathise, Define, Ideate, Prototype and Test steps [28] however the external assessor renamed the Test step “Launch and Share”, to ensure appropriate dissemination of any product or early research findings as per Fig. 1 [24]. Given the importance of evaluating public health interventions yet the lack of robust methodology surrounding those that are co-designed, the Shifra team planned for process evaluation to be undertaken regularly and as objectively as possible.

#### Shifra’s human-centred design process

##### Empathise & define

Empathy sessions between Arabic-speaking refugees, refugee advocates and healthcare workers took place with final year UX students from Monash University throughout the first half of 2017. Researchers undertook CBPR activities and group surveys to better understand barriers and enablers to accessing healthcare for women from these refugee backgrounds. Partner organisation, Multicultural Centre for Women’s Health (MCWH), referred four refugee end users and two others were recruited using snowball-sampling techniques. MCWH also assisted in connecting the CBPR researchers to women interested in helping the Shifra team improve their understanding of the healthcare journey of different women from within these communities in Melbourne.

##### Ideate & design

UX students designed a solution based upon the insights gained during these empathy sessions. These designs were ideated then iterated with end users and other key stakeholders over several sessions throughout Monash University’s first semester in 2017. After selecting the winning design, Shifra’s founder participated in a hackathon hosted by Random Hacks of Kindness, a not-for-profit company that connects business analysts, programmers and UX designers with social impact organisations for weekend long prototyping meetups. Several computer programmers worked on Shifra at the event and continued to develop the technological component of the app until the next hackathon five months later. The beta version of this app was developed, tested and iterated with Arabic-speaking refugees over the next five months.

SMEs vetted health information and simplified content into plain language for accuracy and accessibility. After development of the initial prototype, a more advanced version was user tested with two different groups of co-designers. First, Arabic-speaking women tested the beta version of the app through a partnership with a local adult education program and neighbourhood house, located in Melbourne’s inner-city suburbs where one in four people are from migrant or refugee backgrounds. Again, the app’s content was user tested with SMEs for accuracy and accessibility. Online SMEs from around the world completed functionality and basic content testing via a Qualtrics online survey software and a group of local SMEs then met in person to review and edit the health content, ensuring it was evidence-based before simplifying the information further into plain English prior to Arabic translation. The six people who attended user testing sessions and the all SMEs were recruited using snowball-sampling techniques.

##### Launch & share

Shifra launched the beta version of the app in August 2017 with an event attended by co-designers, supporters, and funders.

## Methods

### Evaluation questions

The evaluation was designed and conducted by an external assessor (JSS) to reduce bias and focused on the following three questions:

1. To what extent did Shifra complete all the steps of the design thinking process shown in Fig. 1?
2. To what extent did the final Shifra app incorporate the contributions of all co-designers?
3. To what extent were the co-designers satisfied with the process?

The first question assisted Shifra’s team to understand how faithful to design thinking principles the initial co-design sessions were. The answer to this question, determined through a scoring rubric, could help the team improve future co-design endeavours. Shifra could only score a full 12 points if it adequately addressed the following criteria:

- End users in co-design sessions were engaged, felt respected, were compensated and were representative of the whole target population
- Empathy exercises were undertaken to understand the lived of experience end users
- Learnings from empathy exercises were compiled, brainstorming solutions sessions were done in teams, end user insights guided the creation of the solution, additional information was gathered from end users if necessary
- Group consensus was obtained on the problem to be addressed/solved. Group consensus was obtained regarding which solutions to prototype to solve the identified problem
- Multiple iterations of prototypes and/or MVPs were created and tested with end user population
- End user feedback was incorporated into subsequent iterations of solution
- Solution was validated with subject matter experts and/or existing literature
- Product was launched
- User testing was completed to understand users’ experience and satisfaction
- User feedback was incorporated into plans for future iterations
- Process or product results were shared with program staff, co-designers, and wider community

A score of < 3 equated with poor evaluation, 4–6 with adequate, 7–9 good and > 10 was equivocal to excellent though feedback should always include room for improvement.

The second question was intended to determine the extent of co-designer involvement, a hallmark of the HCD approach. The third and final question would not only assess co-designer satisfaction but also inform decision-making about how to structure future design sessions.

### Ethics approval

Monash University provided ethics approval prior to data collection (Project ID number 13811: Evaluating the process and product of Shifra’s mHealth intervention). As the external evaluator was from The University of North Carolina-Chapel Hill (UNC), the Institutional Review Board from UNC also reviewed the evaluation plan and determined no additional ethics approval requirements for this project (UNC-Chapel Hill Study #18–1449) Table 1.

**Table 1:** Co-designer involvement

Co-designer category	Process involvement *n* =	Evaluation involvement *n* =	Involvement details
Refugee	6	4	Attended 2 design and 1–2 test sessions.
UX/ Programmer	3	3	Attended all design and test sessions
SME	7	6	Attended 1 design and 1 test session

### Data collection approach

The primary data for the first question (completion of designing thinking steps) involved a thorough review of all of Shifra’s organisational documents including student design reports, community based participatory research (CBPR) results, meeting notes from prototyping events, Qualtrics data from user testing, emails between Shifra staff and the computer programmers regarding app updates and requests, as well as launch event information. Since there is a notable lack of validated tools evaluating HCD projects, a maturity rubric was designed to synthesize the findings from the document review. This rubric was developed through consultation with two experts in the field of implementation science (RR, JAB), two reproductive and indigenous health experts (JAB, RB), one HCD expert (RR) and one participatory research expert (TR). Several iterations of feedback from the expert panel were used to improve the usability, completeness, and level of detail of the rubric (Table 2). While the rubric and design steps used (Fig. 1) appear linear, the design process is fluid and the steps listed did not necessarily occur in a stepwise fashion. The rubric assesses the level of completion of each step of the design thinking process on a scale from 0 (non-existent) to 3 (full completion) with a maximum score possible of 12.

**Table 2:** Shifra HCD evaluation score

	Comments	Points
**Empathise and define**		
**Moderately attempted: (2 points)** End users were engaged, felt respected, and were compensated for their participation in co-design sessions. (Compensation may be financially via cash or gift cards, transportation costs to get to sessions, provision of childcare during sessions, or other means.) Empathy exercises were undertaken to understand lived experience of end users.	• The organisational documents provided evidence of compensation, end user interviews and ethnographic work, CBPR and empathy exercises but there was no documentation regarding whether co-designers felt respected or recognised for their contribution. Instead, this information came from the surveys. • All co-designers selected ‘agree’ or ‘strongly agree’ to feeling respected and if they would encourage others to participate in a co-design session with the Shifra team. • The representativeness of the end user co-designers was also not apparent in any of the organisational documents but emerged as a theme during the interviews. • Co-designers’ views on how representative the end users were varied by the type of co-designer group the participant came from. • SMEs and UX/programmers generally agreed that there was enough end user representation at the co-design sessions. As one computer programmer said: *“We focused bringing on more and more people from the refugee and migrant community which is really good … I don’t think we had a shortage of that diversity … in terms of cultural background it was quite well represented.”* • The end users themselves felt that there were groups within the Arabic-speaking population that were unrepresented. Some of the suggested groups include individuals who did not attend university, Arabic speakers with no or low English proficiency, middle and late middle age individuals, people with different levels of proficiency with mobile technology, and refugees who had just arrived to Australia compared to refugees who have been living in Australia for some time. • A more representative end user population would have accrued a higher score.	2
**Ideate and design**		
**Minimally attempted: (1 point)** Learnings from empathy exercises were compiled. Pre-determined solution was minimally modified in response to what was learned from end users.	• An extensive document review verified that the Shifra team did not have a pre-conceived idea of what the prototype would be, and that the app’s features and structures arose in response to insights gained from the end user co-designers. • The ideation stage took place within the design student teams and was tested at intervals with end users. • Surveys and interviews with the co-designers revealed that one quarter of all survey participants marked ‘neither agree nor disagree’ to whether Shifra confirmed group consensus for either the problem statement or the solutions to be prototyped. The remaining three quarters indicated they ‘agree’ or ‘strongly agree’ that Shifra did in fact, achieve this. • Whilst the issue of consensus did not emerge as a theme across all interviews, one computer programmer articulated the problem this way: “*I feel like sometimes there was a bit of disconnect between what [Shifra staff] wanted and maybe necessarily what the refugees wanted … they wanted a resource where they could find health information, locations specifically of hospitals, GPs, pharmacies … the actual health information, they would rather go directly to the source, rather than … reading it online*.” • Given the conflicting information received from the surveys and the interviews and the lack of documentation, the evaluator reported finding difficulty scoring Shifra in this area. • A lower score of 1 out of 3 was given to draw attention to this issue in the future. • More thorough record keeping during this design stage will shed light on this process and the methods used in future HCD projects.	1
**Prototype**		
**Satisfied: (3 points)** Multiple iterations of prototypes and/or MVPs created and tested with end user population. End user feedback incorporated into subsequent iterations of solution. Solution validated with subject matter experts and/or existing literature.	• End users from different backgrounds tested and contributed to iterations of the app on multiple occasions and continue to do so to this day. • SME co-designers and their feedback lead to tangible changes in the app appearance, language accessibility and functionality. • Organisational documents alone verified these requirements	3
**Launch and share**		
**Satisfied: (3 points)** Product launched. User testing completed to understand users’ experience and satisfaction. User feedback incorporated into plans for future iterations. Process or product results shared with program staff, co-designers, and wider community.	• Shifra held a product release in August 2017 which included co-designers, partners, and funders and presented preliminary findings from the CBPR projects.	3
**Total score (max score of 12):**		9

A survey (Table 3 in Appendix) and a semi-structured interview guide (Table 4 in Appendix) modelled on the IDEO Field Guide to Human Centred Design and mHealth evaluation guidelines [10, 29, 30] were created to answer the second and third evaluation questions as well as to obtain clarification and confirmation of the data obtained through the document review.

These questions explored end user representation, co-designers’ understanding of the co-design sessions and design thinking methods used, issues around communication (i.e., language barriers, role clarification and understanding HCD goals), and co-designers’ levels of satisfaction, using a Likert scale, (Table 3 in Appendix) regarding their involvement in the process.

### Survey and interview procedures

The external evaluator (JSS) engaged three groups of co-designers to complete surveys and semi-structured interviews. All co-designers were approached however not all responded or accepted the invitation to be involved in the evaluation. This included four refugee end users (out of six originally involved in the project), three UX designers or computer programmers (UX/programmers) and six SMEs (including one funding representative) totalling 13 respondents and representing approximately 80% of those involved. All SMEs and UX/programmers were fluent in English even if it was not their native language. End users considered themselves “conversational-level” English speakers. Ten of the respondents identified as women. All co-designers had at least a bachelor’s-level education. Shifra compensated the refugee end users for their time participating in the evaluation through store-bought gift cards.

The co-designers who participated in the evaluation represented the larger co-design groups involved in creating the Shifra app in relation to gender identity, education level, and English proficiency. Interviews with each participating co-designer were consented to in advance and then again in person when they were completed at a location of the co-designer’s choosing. All Arabic-speaking end users declined the use of an interpreter. The survey was administered before the interview, with three exceptions: one SME declined to answer the survey, and two phone interviewees completed the surveys after the interview. The surveys were analysed using Qualtrics online survey software. Interviews were recorded and transcribed. Transcripts were de-identified, coded inductively with a hierarchical framework using NVivo 12 software by the external evaluator and a research assistant (NK), herself a daughter of immigrants, who requested to work on this project because of its migrant women’s health focus.

## Results

Shifra scored 9 out of 12 for fidelity to the design thinking process with a notable need for improvement around the ideation stage (Table 2). Feedback obtained through both the interviews and surveys verified that the Shifra team did complete all the steps of the design thinking approach. All survey respondents selected ‘agree’ or ‘strongly agree’ to statements assessing the collaborative nature of the group work and that they felt safe sharing their opinions (Table 3 in Appendix). Over 90% reported that they would participate in another Shifra co-design session and that they would recommend participation to a friend or family member. In the interviews, a feeling of enjoyment from participating in the co-design sessions clearly emerged. All 13 evaluation participants reported feeling valued, appreciated, and/or respected during the co-design sessions.

… the students, they were very enthusiastic. They take our notes and they try to discuss with us … And as users … they take our notes and they try to improve. And we share, really. We share as a big group, as a teamwork. And we share our ideas together (Source: refugee end user).

The surveys and interviews revealed important learnings for the Shifra team when using HCD.

### Communication

First was the issue of communication. Three of the four end users mentioned that language was a barrier, despite assistance from other community members who acted as interpreters during the co-design sessions and their own self-assessment as being proficient in English. Role clarification was also a need reported by co-designers from all groups. An end user expressed how she came to understand her role in the co-design session:

After some time, I could realise what’s going on, and understand what I had to do. It wasn’t clear in the beginning. Like when I went there, I didn’t know why I’m going there. I just know that I want to be part of this, this is what I really wanted to do, and yeah, after some time I could understand what’s going on, but nobody explained me how.

(Source: refugee end user).

### Fragmentation of involvement

There was fragmentation of co-designers’ experiences during the sessions. Many spoke about not understanding the project’s entire process, wanting to be more involved but not receiving further invitations, or the need to build on previous sessions with end users. While end users were involved at every step of the co-design, different individuals participated at different points and in different ways. Very few end user co-designers were a part of the process from beginning to end. This led to feelings of disconnection and confusion, as one co-designer put it:

First, I was really interested, but after some time when I found like nobody’s calling you back, so I said no I don’t want to waste my time on this. But it’s something really helpful and I really like the idea of helping new arrivals from refugee and migrant backgrounds.

SMEs who facilitated meetings between Shifra staff and end users also felt the desire to be more involved throughout the entire HCD project. One SME commented:

I think it would be good to have a follow up consultation on working on actual usage … because we haven’t sort of touched base again with those women to say, have you used it? … it was almost still in the design stage, and things hadn’t quite been finished. So, I think we could have a follow up that says, this is the latest version of the product, let’s have a play around with it, what do you think now? I think that would be really timely (Source: SME).

A third area for consideration in future co-design session is the importance of diversity and representation within end-user groups. The SMEs and UX/programmer generally agreed that there was sufficient end user representation at the co-design sessions:

We focused bringing on more and more people from the refugee and migrant community which is really good … I don’t think we had a shortage of that diversity … in terms of cultural background it was quite well represented (Source: UX/programmer).

The end users themselves, however, felt that there were groups within the Arabic-speaking population that were unrepresented. Some of the suggested groups include individuals who did not attend university, Arabic speakers with no or low English proficiency, middle and late middle age individuals, people with different levels of proficiency with mobile technology, and refugees who had just arrived to Australia compared to refugees who have been living in Australia for some time. The risks of only collaborating with end users who are university-educated, recently resettled and English-proficient were summarised by one end user:

Maybe we will use this program Shifra and maybe never we will use this program because we can search … what we need by Google … but that program Shifra, it’s good for different level of the people (refugee end user).

Increased representation from within the end-user group should be considered for future HCD endeavours.

Finally, a survey question assessed whether co-designers felt that there was enough time for relationship building during the co-design sessions. End users responded most negatively to the statement, with half stating that they ‘strongly disagree’ or ‘disagree’ that there was enough time for this.

## Discussion

This evaluation found that a co-design process was successfully applied to the development of a web-based app for refugee and migrant women in reproductive health. This evaluation also yielded several important recommendations for improving Shifra’s HCD approach moving forward, findings that can be applied to other projects seeking to undertake an authentic community co-design process. First, with so many people of diverse backgrounds contributing to the project, clear communication about roles and expectations is critical. More attention to facilitator training, identification of session goals, following up with consistent communication, and seeking end user and SME feedback would help to reduce future confusion [10]. Second, it is important to set realistic expectations and role clarifications with co-designers. Design is a non-linear and creative process, which can inadvertently contribute to confusion about the co-designer’s purpose and the project’s goals [31]. Care should be taken in advance to explain this and answer questions from participants not familiar with the concept.

Third, it is important not to view all end users as interchangeable [10]. UX/programmers and some SMEs saw refugees at all the meetings and viewed that as enough end user participation. End users however, felt there were other voices from their community that needed engagement. For example, the fact that all the end users spoke some English meant that co-design sessions could proceed without certified interpreters, but it also meant that the voices and experiences of refugees with low English proficiency were missing. There is also the issue of inherent bias. As mentioned, one refugee end user’s opinion was dismissed by a designer until it was supported by an SME. This was not tolerated by the Shifra team and the designer’s supervisor was notified to mentor the designer in question. Ultimately though, this shows how HCD in and of itself can be flawed and active steps need to be taken to reduce, and where possible remove, power structures that pervade everyday life. Sasha Costanza-Chock’s (2020) notes that “Design justice asks whether the affordances of a designed object or system disproportionally reduce opportunities for already oppressed groups of people while enhancing the life opportunities of dominant groups, independently of whether designers intend this outcome.” [32] Increasing the influence of end users needs a purposeful and planned approach, one that most projects, including Shifra’s, needs to improve upon in the future.

There will naturally be trade-offs in any public health project since limited resources are an unchanging reality, but several process adjustments could address this issue. This evaluation demonstrates that the practice of engaging end users across all empathy, design, and prototyping stages and into product development is possible [31]. Collaborating with multiple migrant and refugee advocate organisations to use diverse sampling techniques will help to engage a more representative sample in the future co-design sessions.

Finally, by setting aside adequate time to develop collaborative relationships amongst all co-design groups the HCD process is an opportunity to give power and control back to the end user population for whom one is designing the health intervention [33]. Placing greater effort into building relationships as a part of the co-design session is especially important with Shifra’s partner refugee communities [26]. When properly implemented, the intent of HCD is to provide public health organisations a pathway to sharing (and where applicable, handing over) power in order to achieve true citizen participation and control [25]. Failure to apply HCD principles in an authentic or purposeful way usually results in tokenism, and development of solutions that are unsustainable [33]. It can also “exacerbate social exclusion and destroy trust systems” when done poorly [34]. A project cannot utilise HCD without a power dynamic shift that ensures the end user, not the UX designer, computer programmer or community organisation, is in the primary decision-making role [11].

### Future considerations

There are several important considerations when planning for evaluation of any HCD-driven projects. First, while this version of the rubric was helpful in gaining a deeper understanding of Shifra’s HCD approach, several iterations will increase the tool’s usefulness moving forward. Including operational definitions of each of the three stages would clarify the expectations, especially for anyone who is trying to use the rubric to guide future HCD-driven projects. Additionally, some of the requirements were impossible to assess using existing documentation alone. For example, the evaluator was unable to ascertain whether end users felt respected from organisational documents alone and all three data sources were required to complete the rubric. As mentioned, future papers will assess the influence or impact of the app on user SRH literacy and determine the relative success of the health intervention. Questions specific to the user’s experience of the app may demonstrate areas where refugee voices came through strongest and others where their perspectives needed to be elevated more. Likewise, co-designers’ perceptions of whether they felt respected and valued during co-design sessions should be included all future feedback forms, surveys and interview guides. This is especially important in helping reduce implicit bias within the design process that may be overlooked or go unacknowledged due to inherent power structures reinforced by unchecked privilege, however unintentional they may be.

Using multiple data sources (document review, surveys, and interviews) should have helped to verify answers. Instead, each data source provided unique and isolated findings and there proved inconsistency between interviews and the survey responses concerning communication. For example, while the majority (75%) of co-designers responded ‘agree’ or ‘strongly agree’ to the survey statement about whether roles and responsibilities had been clearly explained, many anecdotes arose during the interviews around the fact that co-designers did not actually understand their role in the overall project or at specific co-design sessions. Similarly, one of the main themes of the semi-structured interviews was how fragmented the co-designers felt their involvement was though there was no way to verify this finding in the document review or with the surveys. One way to address this problem is by considering evaluation methods alongside HCD planning meetings. Collecting co-designer surveys throughout the project can help staff understand their experiences ‘in the moment’ and offers an opportunity for a more agile response if needed, whilst also offering an opportunity to compare experiences and feedback later following end of project co-designer evaluations. Shifra did not have any documentation regarding training materials on design thinking facilitation or how facilitators had introduced the co-designers to various methodologies at different points in the app’s development, making it difficult to verify these findings. Future documentation on roles, design thinking goals and checking in with co-designers frequently would benefit all involved.

### Strengths and limitations

This paper adds to the existing literature regarding the rigorous use of HCD in public health. This implementation evaluation provides an important guide to purposefully working with end user communities to design better health interventions. Engaging in evaluation work increases the transparency of organisations and helps them demonstrate their commitment to the HCD ethos. This paper and the rubric are helpful tools for organisations attempting to evaluate their use of design thinking methods, and can assist them to plan, prepare for, and execute successful co-design sessions. Additionally, the evaluation included multiple sources of data including surveys, interviews, and records. While Shifra’s data collection methods will be improved as a result of this evaluation, multiple sources of data give a more full, rich, and accurate picture of co-designers’ experiences and the methods themselves [35]. The richness of the data obtained is especially important since HCD is nebulous by nature and there is a lack of demonstrated, rigorous evaluation [20, 36]. Finally, the utilisation of an external assessment increased the objectivity of this evaluation [37].

There were several limitations to this evaluation study. First, the small sample size and convenience sampling of co-designers introduced selection bias into the results. It is possible that the co-designers who did not respond to a request to participate in the evaluation would have provided different answers and perspectives. There were only four end users involved in this evaluation, limiting the feedback and perspectives of those most important to both the mission and process being undertaken in developing the Shifra app. Second, the evaluation took place 9–16 months after most of the co-design sessions, making results vulnerable to recall bias. Several interviewees mentioned at different points that the sessions had happened so long ago it took effort to remember and answer the questions. Third, a yet to be validated rubric guided part of the evaluation. Despite expert feedback and iteration on this rubric before its application, there needs to be more use of the checklist to ensure that it is both reliable and generalisable. Fourth, within the research team, only the research assistant (NK) had any personal connection to the migrant or refugee experience. Future research on migrant or refugee communities must include funding to support people from migrant and refugee backgrounds to play an active, and ideally lead, role in designing, conducting, analysing and reporting on said research. Finally, evaluating HCD, design thinking and co-design efforts in public health is challenging due to its abstract, creative, and iterative nature and because results are specific to the local context. There are no definitive guidelines providing specific parameters for assessing a HCD-driven project and these terminologies are frequently interchanged despite their differences in application and outcome. The methods identified in this paper are a first attempt to benchmark this innovative approach and will need to be refined in the future.

## Conclusion

Improving refugees’ access to sexual and reproductive health is complex and multidimensional and requires innovative and thoughtful problem solving. HCD is one way to address complex problems in, ideally, a more ethical and effective way and it is how Shifra chose to approach the development of its solution to this problem among Arabic-speaking refugees in Melbourne, Australia. The surveys and interviews revealed that end user, SME, and UX/programmer co-designers enjoyed participating in the co-design sessions, felt respected and welcomed, and saw their contributions reflected in the final product. Opportunities for growth include engaging a more diverse end user population and communicating expectations and results more clearly during and after the co-design sessions. A comprehensive process evaluation benefits the field by providing an example of how to assess an organisation’s ability to follow all the HCD steps based on individualised contexts *and* will advance knowledge on the effectiveness of HCD in developing solutions that are aligned with the needs of the target audience. It also provides an opportunity to explore implicit bias and inherent power structures present in HCD methodologies that may be unintentional and may, therefore, go unchecked. This evaluation of Shifra’s HCD approach provides a helpful and rigorous guide in reporting that may encourage other organisations undertaking HCD work to evaluate their own implementation. Such organisations should explore in advance, how they plan to evaluate not only the design steps but also co-designers’ perceptions around their role and the contributions they made to the end product. Determining how to assess satisfaction with both process and product needs thoughtful consideration to ensure co-designers and evaluators are reflecting and measuring the same outcome respectively. Finally, utilising mixed methodologies has the potential to reveal inconsistent answers across the different sources of data being examined so care needs to be taken to ensure evaluative and probing questions in the semi-structured interview process, add clarity and reduce confusion.

## Data Availability

The data that support the findings of this study are available from author, JSS, but restrictions apply to the availability of these data, which were used under license for the current study, and so are not publicly available. Data are however available from the authors upon reasonable request and with permission of author, JSS.

## Appendix

**Table 3:** Participant Survey Feedback Form

At the co-design session …		Strongly disagree	Disagree	Neither disagree nor agree	Agree	Strongly agree	N/A
1	The group work felt collaborative						
2	My contribution was valued						
3	People attended who do not usually have representation (i.e. people with refugee and migrant backgrounds, people with diverse sexual orientation, people experiencing health accessibility issues)						
4	The voices of refugee and/or migrant end users were heard						
5	The roles and responsibilities of my participation were clearly defined						
6	I understood both the processes and the language used						
7	My time participating was compensated appropriately						
8	There was a commitment by Shifra staff to develop consensus on what the end product should include						
9	All co-designers were kept informed of any changes						
10	I felt respected by Shifra staff						
11	I felt respected by all co-design partners						
12	Shifra staff offered me an opportunity for skill development and capability building						
13	Shifra staff offered me co-design training and resources						
14	There were strategies to involve people with different communication needs						
15	There was enough time to allow relationship building						
16	Shifra staff made attempts to reduce any power imbalance (e.g. between health professionals and refugees)						
17	I felt safe sharing my opinions						
18	Refugee and/or migrant end users helped shape the common agenda						
19	I would participate in another co-design session with Shifra staff						
20	I would encourage others to participate in a co-design session with Shifra staff						
21	I see my ideas and contributions reflected in the final Shifra website/app						

Comments/Feedback:

**Table 4:** Semi-structured interview guide

1	Could you elaborate on how you felt your contribution to the co-design sessions was (or was not) recognised and valued?
2	Were there stakeholders who were not represented at the co-design sessions? If so, who else should have been invited?
3	Was there any confusion regarding your role, the processes or any language barriers at the co-design sessions that could have been made simpler/easier to understand? If so, what were these?
4	Were there ways that the Shifra team or other participants made you feel respected or disrespected/unwelcome during this co-design experience?
5	Do you feel that any power imbalances between co-design participants were addressed e.g. do you feel that your opinion mattered and that it was safe for you to communicate your thoughts and experiences if you wanted to?
6	How could this co-design experience be improved in the future?
7	Do you have any other feedback you wish to share?

## Abbreviations

CBPR: Community based participatory research
HCD: Human centred design
MCWH: Multicultural Centre for Women’s Health
SRH: Sexual and reproductive health
SME: Subject matter experts
UNC: University of North Carolina
UX: User experience
